# Mental Health Nurses’ Perspectives on Cognitive Behavioural Therapy for Individuals Diagnosed with Schizophrenia in Saudi Arabia: A Qualitative Study

**DOI:** 10.3390/healthcare14172852

**Published:** 2026-09-04

**Authors:** Muteb Aljuhani, Karina Lovell, Owen Price

**Affiliations:** 1 Department of Community, Mental Health, and Psychiatric Nursing, Imam Mohammad Ibn Saud Islamic University (IMSIU), Riyadh 11564, Saudi Arabia; 2 Department of Nursing Midwifery and Social Work, The University of Manchester, Manchester M13 9PL, UK

**Keywords:** cognitive behavioural therapy, mental health nurses, schizophrenia, Saudi Arabia, qualitative study

## Abstract

Background/Objectives: Cognitive behavioural therapy (CBT) is an effective intervention for promoting personal recovery among individuals diagnosed with schizophrenia; however, Saudi Arabia has a shortage of healthcare professionals trained to deliver it. Mental health nurses are well positioned to help address this gap. This study explored mental health nurses’ perspectives on a proposed culturally adapted CBT intervention for individuals diagnosed with schizophrenia and identified perceived barriers and facilitators to its implementation. Methods: Situated within the development phase of the Medical Research Council framework for complex interventions, the study examined perceived acceptability and anticipated feasibility rather than efficacy. Three face-to-face focus groups involving 19 mental health nurses were conducted between December 2020 and February 2021 at a tertiary mental health hospital in Riyadh, Saudi Arabia, during the COVID-19 pandemic. The focus group questions were informed by a systematic review of culturally adapted CBT components, and data were analysed using framework analysis. Results: Three themes emerged: acceptability, with nurses recognising the value of CBT in promoting recovery; barriers, including lack of role clarity within mental health nursing policy, dominance of the medical model, and engagement difficulties among individuals diagnosed with schizophrenia; and facilitators, including supportive nursing management, training opportunities, and family involvement. Conclusions: Mental health nurses expressed support for delivering CBT but identified organisational and training-related barriers. Successful implementation will require clear policy frameworks, structured culturally adapted CBT training, ongoing clinical supervision, supportive nursing management, and collaborative approaches that appropriately involve families.

## 1. Introduction

Schizophrenia is a serious mental illness characterised by disorganised thought patterns, catatonic behaviour, and a spectrum of positive and negative symptoms [1]. Although its underlying aetiology remains unclear, numerous factors have been associated with an increased risk of developing the illness. These include genetic predisposition, migration-related stress, exposure to trauma, and prenatal infections [2,3,4]. Given its multifactorial aetiology, treatment approaches include antipsychotic medication and psychosocial interventions, such as cognitive behavioural therapy (CBT) [5].

CBT is an effective psychosocial intervention that can facilitate personal recovery in individuals diagnosed with schizophrenia and is formally recommended by the National Institute for Health and Care Excellence as part of standard treatment guidelines [5]. Evidence indicates that CBT can reduce the positive and negative symptoms of schizophrenia [6,7,8]. Although CBT has demonstrated effectiveness in Western contexts, its effectiveness in non-Western contexts may be less apparent when insufficient attention is given to cultural adaptation during the early stages of intervention development [9]. Consequently, cultural adaptation of CBT is strongly recommended before implementation in non-Western contexts such as Saudi Arabia [9]. Schizophrenia constitutes a substantial component of the mental health burden in Saudi Arabia. In a multi-region study of 1205 patients across six hospitals, schizophrenia was the most common psychiatric diagnosis overall (38.8%) and among inpatients (55.8%) [10]. This concentration of schizophrenia within Saudi psychiatric services, particularly inpatient care, underscores the need for accessible, evidence-based psychological interventions for this population.

Within Saudi Arabian mental health services, psychosocial interventions are predominantly delivered by psychiatrists and psychologists. However, the availability of these professionals is limited, with 1.32 psychiatrists and 2.03 psychologists per 100,000 population [11]. These rates are substantially lower than those in the United Kingdom, where there are 14.63 psychiatrists and 12.83 psychologists per 100,000 population [11]. By contrast, Saudi Arabia has 10.66 mental health nurses per 100,000 population [11].

Mental health nurses are therefore well positioned to help address this workforce shortage and contribute to the delivery of culturally appropriate CBT interventions in Saudi Arabia. Beyond any potential role in delivering CBT, mental health nurses provide much of the continuous, front-line care within mental health services, where the therapeutic relationship is central to their work with individuals experiencing psychosis [12]. Their responsibilities include skilled assessment of individual needs, risk identification, medication administration and monitoring, therapeutic engagement, psychoeducation to support self-management, and collaborative work with families and the wider treatment team [12]. This sustained contact places mental health nurses in an advantageous position to incorporate structured psychological interventions such as CBT into routine care and has prompted calls to advance mental health nursing practice for people with psychosis in Saudi Arabia [12]. Evidence indicates that mental health nurses can be trained effectively to deliver CBT to individuals diagnosed with schizophrenia [13,14,15,16,17]. For example, randomised controlled trials have shown that mental health nurses trained and supervised in brief CBT can achieve meaningful improvements in symptoms and insight among individuals diagnosed with schizophrenia [15,18]. These findings suggest that, with appropriate training and supervision, mental health nurses can deliver CBT safely and effectively to individuals diagnosed with schizophrenia [18].

Although cultural adaptation of CBT for psychosis has been examined qualitatively in other contexts, for example through studies exploring the views of patients, carers, and mental health professionals in Pakistan and China [19,20,21], these studies did not focus specifically on mental health nurses. In Saudi Arabia, the preceding phase of the present programme similarly examined the perspectives of individuals diagnosed with schizophrenia and their family members [22].

The perspectives of mental health nurses on delivering CBT for schizophrenia in Saudi Arabia have not previously been investigated. Mental health nurses differ from psychiatrists and psychologists because they provide much of the continuous, front-line contact and are particularly well positioned to deliver a scalable, task-shared intervention. The present study addresses this gap.

This study aimed to: (1) explore mental health nurses’ perceptions regarding the delivery of a proposed culturally adapted CBT intervention for individuals diagnosed with schizophrenia in Saudi Arabia; (2) identify the perceived barriers and facilitators to nurse-delivered CBT in this context; and (3) inform the subsequent development of the culturally adapted CBT intervention within the Medical Research Council framework for complex interventions.

## 2. Materials and Methods

This study formed part of a PhD thesis, completed in 2022, that aimed to develop a CBT intervention for delivery by mental health nurses to individuals diagnosed with schizophrenia in Saudi Arabia [23]. The studies comprising this programme of work are being published sequentially. The perspectives of individuals diagnosed with schizophrenia and their family members were reported in the first paper [22], whereas the present paper reports the second phase, focusing on mental health nurses. The research was guided by the Medical Research Council framework for complex interventions [24].

In the first phase, the perspectives of individuals diagnosed with schizophrenia and their family members on the proposed CBT intervention were explored [22]. Most participants accepted the proposed intervention and identified therapist characteristics, family involvement, religion, and the number and format of sessions as key influences on engagement and motivation [22]. These findings directly shaped the present phase by indicating the need to examine perceived acceptability; anticipated feasibility of delivery should also be examined from the perspective of mental health nurses, the professional group best positioned to help address the workforce shortage. They also informed the focus group questions, which explored nurses’ views on the acceptability of the proposed CBT components, family involvement, strategies to support engagement, session structure, and the organisational barriers and facilitators to intervention delivery. The present study constituted phase two and used qualitative methods, which are particularly valuable during the early stages of psychosocial intervention development [24]. This developmental approach was also informed by evidence suggesting that insufficient attention to intervention development can adversely affect engagement and apparent effectiveness.

For example, Wahass and Kent [25] evaluated CBT in Saudi Arabia and reported low levels of participant engagement and retention, which they attributed to insufficient attention to the intervention-development phase. Accordingly, before proceeding to a randomised controlled trial, the present programme adopted a cautious development-phase approach. This involved a qualitative study of mental health nurses’ views on the proposed CBT intervention for individuals diagnosed with schizophrenia and the perceived barriers and facilitators to its implementation. These methods were chosen to align with the study objectives: focus groups were used to explore mental health nurses’ perceptions of the proposed intervention (Objective 1) and, through group interaction, to elicit perceived barriers and facilitators to nurse-delivered CBT (Objective 2). Framework analysis, an applied and policy-oriented approach, was then used to systematically organise these accounts into actionable barriers and facilitators that could inform the subsequent development of the intervention (Objective 3). The study was reported in accordance with the Standards for Reporting Qualitative Research (SRQR) checklist (see Supplementary File S1 [26]).

### 2.1. Data Collection

Focus groups were used to facilitate interaction among participants, encourage the exchange of experiences and perspectives, and generate in-depth insights into barriers and facilitators to delivering CBT interventions [27]. The discussions were moderated by the lead author, who is trained in focus group facilitation. The lead author conducted and audio-recorded all focus group discussions and transcribed them verbatim in Arabic; he is the only bilingual (Arabic–English) member of the research team. He then translated the transcripts into English, prioritising conceptual and cultural equivalence over literal word-for-word correspondence to preserve culturally specific meanings and idiomatic expressions. To verify translation accuracy, an independent bilingual reviewer with relevant subject-matter expertise who was not a member of the study team checked the English translations against the original Arabic transcripts; any discrepancies were discussed and resolved by consensus. Because the remaining research-team members work in English, coding and analysis were conducted using the English translations. However, as a bilingual native Arabic speaker who had collected the data, the lead author retained and consulted the original Arabic recordings and transcripts throughout the analysis to ensure that coding remained faithful to participants’ intended meanings. The topic guide was informed by the systematic review by Degnan et al. [28], which identified commonly adapted components of CBT for schizophrenia, including engagement and assessment, psychoeducation, management of psychotic symptoms, medication adherence, and relapse prevention.

The focus group questions were designed to explore mental health nurses’ perspectives on the proposed CBT intervention for individuals diagnosed with schizophrenia and to identify potential barriers and facilitators to its implementation (see Supplementary File S2).

### 2.2. Setting of the Study

Participants were recruited from among nursing staff working across inpatient and outpatient units at the Eradah Complex for Mental Health in Riyadh, Saudi Arabia. This 625-bed mental health facility operates through an integrated model encompassing mental health and addiction diagnosis, treatment, and research activities [29]. The site was purposively selected because it is the principal tertiary referral centre for mental health services in the Riyadh region and provides access to a large and diverse population of registered mental health nurses working with individuals diagnosed with schizophrenia across inpatient and outpatient settings. It was therefore considered an appropriate setting in which to explore the study aims. Data collection occurred during the COVID-19 pandemic, which influenced both the sampling approach and the mode of contact, as detailed below.

### 2.3. Sampling

Convenience sampling was used instead of purposive sampling because COVID-19 restrictions limited access to the study site. Pandemic-related infection-control measures restricted on-site access and the researcher’s ability to preselect participants purposively using a sampling matrix or to undertake formal maximum-variation sampling. Recruitment therefore depended on nurses who were available and willing to participate within these constraints. Nevertheless, the achieved sample included variation across relevant characteristics, including years since qualification (2–18 years), gender, staff and charge-nurse grades, qualification level (six diplomas, 12 bachelor’s degrees, and one master’s degree), and service areas, providing a reasonable range of perspectives despite the non-probability sampling approach. None of the participants had received formal CBT training, consistent with the training gap identified in the findings. Eligible participants were registered mental health nurses with at least 12 months of clinical experience who were currently working with individuals diagnosed with schizophrenia in either inpatient or outpatient settings.

### 2.4. Recruitment

The lead author met with the head of nursing to discuss the study and facilitate dissemination of recruitment information. The lead author’s contact details were distributed to wards in which eligible nursing staff worked. Nurses who were interested in participating contacted the lead author directly, and a mutually convenient date and time were arranged. Recruitment took place between December 2020 and February 2021. Participants within each focus group were professional colleagues who, in most cases, knew one another. No non-participant observers were present during the discussions.

### 2.5. Data Analysis

Data were analysed using framework analysis, which comprises five stages: familiarisation, developing a thematic framework, indexing, charting, and mapping and interpretation [30]. During familiarisation, the lead author transcribed the focus group discussions verbatim in Arabic and translated them into English, thereby facilitating early immersion in the dataset. Coding was conducted using the English-language transcripts. The lead author developed an initial coding framework, which was discussed and refined with the co-authors. During indexing, the lead author coded all transcripts in NVivo 12 according to the agreed framework. A codebook containing code labels, definitions, and illustrative excerpts was developed during framework construction and refined iteratively as coding progressed. An audit trail documenting analytical decisions, framework revisions, and the mapping of codes to sub-themes and themes was maintained throughout. The co-authors did not independently code a duplicate set of transcripts; therefore, a numerical inter-coder agreement rate was not calculated. Instead, coding decisions, theme boundaries, and alternative interpretations were reviewed during regular research-team meetings until consensus was reached. During charting, data were summarised in a matrix organised by themes, sub-themes, and focus groups. Finally, the research team reviewed the analytical charts, refined or combined categories where necessary, and mapped relationships across the dataset to produce the final interpretation.

### 2.6. Rigour

Rigour was supported by Lincoln and Guba’s principles of credibility, transferability, dependability, and confirmability [31]. Peer debriefing and regular research-team meetings enhanced credibility by providing critical feedback and consideration of alternative interpretations. Transferability and dependability were supported through detailed reporting of the research purpose, data-collection and analysis procedures, and participant-selection processes. Confirmability was enhanced through the inclusion of direct participant quotations and maintenance of a reflexive journal to mitigate researcher bias. Formal member checking (returning transcripts or findings to participants for verification) was not undertaken. It is one of several strategies for enhancing credibility rather than a methodological requirement, and its value in cross-case interpretive analysis has been questioned [32]. Instead, credibility was supported through complementary strategies appropriate to the study aims: peer debriefing and regular research-team review of coding and interpretation; independent bilingual verification of the translated transcripts; repeated consultation of the original Arabic recordings and transcripts during analysis; and reflexive journalling. Together, these strategies helped ensure that the findings remained grounded in participants’ accounts.

### 2.7. Reflexivity

Consistent with SRQR guidance on researcher characteristics and reflexivity, we explicitly describe the position of the lead author, who conducted the focus groups, transcribed and translated the data, and led the analysis. The lead author was a male Saudi mental health nurse with seven years of clinical experience, including inpatient work with individuals diagnosed with schizophrenia and dual diagnoses. He had no prior professional or personal relationship with the participating nurses. As a mental health nurse, he shared a professional and cultural frame of reference with participants, which facilitated rapport, culturally attuned moderation, and interpretation of idiomatic and culturally specific expressions. Because he was not employed at the study site and had no prior relationship with participants, the potential for hierarchical or supervisory power imbalances was reduced. However, his shared disciplinary background also created a risk that common professional assumptions could influence question framing and coding. To mitigate this risk, the lead author maintained a reflexive journal and bracketed prior assumptions during analysis. Additionally, the coding framework, theme boundaries, and interpretations were subjected to critical scrutiny by the co-authors, who are based outside Saudi Arabia and contributed an outsider perspective.

## 3. Results

Three focus groups, each comprising six to eight mental health nurses, were conducted between December 2020 and February 2021. Each session was held in a quiet room and lasted 60–90 min. The number of focus groups was determined by data saturation, operationalised as the point at which successive groups yielded no new codes, sub-themes, or themes and existing categories were repeatedly confirmed rather than expanded [33]. The research team monitored the emergence of new codes after each focus group. The third focus group generated no new codes or sub-themes and served only to reinforce patterns identified in the first two groups, indicating that saturation had been reached. This is consistent with evidence that a small number of focus groups is typically sufficient to capture the most prevalent themes in a relatively homogeneous sample [33]. Table 1 summarises participant characteristics.

Three key themes emerged from the analysis: the perceived acceptability of the CBT intervention, the challenges associated with its implementation, and the enablers that facilitate its delivery (Table 2).

### 3.1. Theme 1: Acceptability of the CBT Intervention

This theme examines mental health nurses’ views on the suitability of the initial components of the CBT intervention and their suggestions for refining these components to better align with the needs of individuals diagnosed with schizophrenia. Nurses’ openness to CBT was genuine but conditional: acceptance coexisted with uncertainty about whether CBT delivery fell within their professional role, suggesting that willingness alone may not translate into practice without a clearer nursing mandate. The theme comprises one sub-theme, ‘key elements of the CBT approach’, described below.

#### Key Elements of the CBT Approach

Most participants considered the components of the proposed CBT intervention comprehensive and potentially beneficial for individuals diagnosed with schizophrenia. They emphasised that the structured nature of the intervention could support the development of therapeutic relationships, particularly during the initial engagement and assessment phase. This phase was viewed as an important opportunity for mental health nurses to listen actively to individuals’ concerns and support them in managing or resolving their difficulties.

*‘These components, in my opinion, will help people who have schizophrenia deal with their symptoms’.*

(P6, Group 2)

Participants also recognised the critical role of the engagement and assessment phase, noting that the overall effectiveness of the programme could depend heavily on the success of this initial stage. They recommended keeping the assessment process brief and straightforward because lengthy or complex assessments may contribute to disengagement and reduce participation in the intervention.

*‘The patient’s ability to hear and respond to a lot of questions during the assessment phase is limited, as my colleague said, because some of them may become bored. As a result, if the assessment is brief, that would be better’.*

(P16, Group 1)

Participants also considered the medication component particularly important. Drawing on their clinical experience of promoting medication adherence among individuals diagnosed with schizophrenia, they proposed several strategies to support adherence, including medication reminders using mobile-phone alarms.

*‘One patient in the outpatient unit complained about forgetting to take his medication on time, and we suggested he set an alarm on his phone to remind him to take his medication. At his next appointment, he reported that this technique was working’.*

(P12, Group 3)

Participants considered the remaining components of the CBT intervention, including work on psychotic symptoms and relapse prevention, appropriate and did not recommend specific modifications. However, to enhance engagement and motivation and support completion of CBT sessions, they suggested using more accessible and engaging methods. As one participant explained:

*‘Some patients become bored and unable to sit for long periods when listening to a topic. They prefer to watch a video, which allows them to see something practical rather than just hearing about it verbally and so improves their understanding of the topic. For example, in the CBT programme, provide a brief video or colourful booklets to clarify the information you want to deliver to the patients in addition to the verbal explanation’.*

(P19, Group 1)

### 3.2. Theme 2: Challenges to CBT Implementation

This theme explores the barriers and challenges that may hinder mental health nurses when delivering CBT interventions. The principal obstacles identified were structural rather than individual knowledge deficits. Specifically, nurses located the barriers in constraints within mental health nursing policy and the dominance of the medical model, highlighting the organisational and hierarchical context as the main impediment to a therapeutic nursing role.

#### 3.2.1. Constraints Within Mental Health Nursing Policies

One of the main barriers to implementing CBT by mental health nurses was the absence of a clearly defined mental health nursing policy. Despite participants’ varied educational backgrounds (diploma, bachelor’s, and master’s degrees), most were unfamiliar with their specific roles within mental health nursing policy.

Participants reported that their duties in both inpatient and outpatient settings were largely confined to fundamental tasks, including monitoring vital signs, observing the behaviour of individuals diagnosed with schizophrenia, assisting with hygiene, and making beds, all of which are functions typically performed by healthcare assistants. The following quotation illustrates this perceived role:

*‘As a mental health nurse in our outpatient department, I do not have any role to play as a therapist. My role is to just take vital signs or be with the psychiatrist. If CBT isn’t an essential part of the job description, I’m not obliged to do it, which means I don’t have the authorization to do it’.*

(P5, Group 1)

Moreover, lack of clarity regarding the scope of mental health nursing practice led participants to feel marginalised within the mental healthcare system. This ambiguity had several perceived consequences. Participants reported being assigned responsibilities beyond their professional role, including duties typically associated with security personnel or social workers, which they felt increased workload and contributed to burnout. They also described frustration at limited opportunities to deliver psychosocial interventions, particularly among nurses working in inpatient settings.

*‘In the inpatient departments, we have an issue; there are things we do as mental health nurses, but it isn’t a nursing job; for example, I am responsible for opening and closing the department’s doors. I am responsible for making the patient make contact with his family, so if I take a CBT training course, I guarantee that I will not implement it because I am too busy with non-mental health nursing tasks. There are a lot of things imposed on mental health nurses that are not related to our job that keep us very busy’.*

(P3, Group 2)

#### 3.2.2. Predominance of the Medical Model

The medical model, which prioritises symptom management through antipsychotic medication, was perceived to dominate mental health practice in Saudi Arabia. Participants described a predominant reliance on pharmacological treatment and the centralised authority of psychiatrists in formulating treatment plans for individuals diagnosed with schizophrenia. In outpatient settings, they reported that pharmacological treatment was often the only option offered. Participants also perceived limited confidence among psychiatrists in the effectiveness of psychological interventions and reported that referrals to psychologists were uncommon. Consequently, they believed that the dominance of the medical model would present a substantial barrier to implementing CBT interventions.

*‘It is almost certain that the doctors play the most important role, which means that when they meet with patients, they do not refer them to a psychologist but instead prescribe medications for them, even if their condition is minor and does not require medication. This is an incorrect approach that has been practised for years, implying that there is no education or intervention in place in the outpatient unit’.*

(P7, Group 3)

Notably, although participants expressed concern that the medical model might hinder the implementation of nurse-delivered CBT, some of their reported practices and attitudes were themselves consistent with this model. Several participants viewed medication adherence as the most effective means of supporting individuals diagnosed with schizophrenia and described a primary nursing role centred on ensuring adherence to prescribed medication regimens. Some also suggested that physical restraint might be necessary when individuals refused medication.

*‘The nurse talks to the patient and informs him that his disease is chronic and that he should not stop taking his medications; if he does, the nurse will physically restrain him again. This procedure will allow the patient to continue taking his prescription medications and not stop taking them when he leaves the hospital’.*

(P2, Group 1)

### 3.3. Theme 3: Enablers of CBT Implementation

This theme presents the strategies and recommendations proposed by participants to address barriers to CBT implementation. Nurses framed the solutions as organisational and relational rather than solely individual, emphasising supportive nursing management, structured training, and family involvement as key conditions for embedding CBT in practice.

#### 3.3.1. Mental Health Nursing Management

Participants emphasised the central role of mental health nursing management in facilitating CBT implementation. They noted that nursing management has the authority and responsibility to establish mental health nursing policies and define the scope of nursing practice within psychiatric care settings. Participants believed that defining the scope of practice in accordance with nurses’ qualifications could reduce inappropriate workload and enable more effective delivery of CBT interventions. Some also highlighted the broader problem of mental health nurses being assigned tasks outside the scope of mental healthcare.

*‘Regarding the CBT intervention, the nursing administration has a significant role. They have the authority to add the CBT intervention to the nursing policy and nominate the person who will deliver the intervention, thus playing a significant part in ensuring that the CBT intervention is implemented successfully’.*

(P5, Group 1)

Participants also emphasised the importance of coordination with the wider healthcare team, including psychiatrists, psychologists, and social workers, in supporting successful implementation of the CBT intervention. They viewed such coordination as a key responsibility of mental health nursing management. In particular, they considered this coordination necessary to secure approval from relevant hospital authorities for mental health nurses to deliver CBT and to align CBT sessions with other professionals’ schedules and treatment plans, thereby reducing conflicts and promoting integrated care.

*‘The healthcare team can refuse to allow mental health nurses to offer this programme, which implies that the mental health nursing administration must first obtain the healthcare team’s consent and then incorporate CBT into the mental health nursing policy’.*

(P13, Group 3)

#### 3.3.2. Availability of Training Opportunities

Effective delivery of a CBT intervention for individuals diagnosed with schizophrenia depends substantially on provider knowledge and competence. Participants regarded comprehensive training as essential and emphasised the need for ongoing supervision to ensure that mental health nurses were adequately prepared to deliver the intervention confidently and effectively.

*‘As a mental health nurse, I should have the required training to offer the CBT intervention. We have many departments; we are ready, but we lack the tools. I mean, the knowledge, skills, supervision, and plan for how to offer the CBT intervention’.*

(P15, Group 3)

Participants also emphasised that training should address the broad range of clinical presentations among individuals diagnosed with schizophrenia, particularly complex presentations that could challenge intervention delivery. For example, they highlighted the need for strategies to work with individuals experiencing persecutory delusions involving the nurse delivering the intervention and with those experiencing command hallucinations.

*‘We need someone who specializes in CBT to train nurses qualified for the programme. There are things we want to learn, such as how to manage patients with various symptoms; thus, we need training to feel more confident when implementing the programme. For example, how do I deal with patients who have a persecutory delusion about me?’*

(P6, Group 2)

Although most participants were eager to implement the CBT intervention, some suggested that training should be prioritised for mental health nurses who demonstrate genuine interest and motivation to deliver it. They noted that some nurses were resistant to professional development and frequently declined training opportunities offered by mental health nursing education departments.

*‘The mental health nurses who will be nominated to offer the CBT intervention must have a strong desire to do so’.*

(P1, Group 2)

#### 3.3.3. Active Involvement of Family Members in Care

Participants considered family involvement an important contributor to the success of the CBT intervention. They noted that most individuals diagnosed with schizophrenia live with family members and are often accompanied by relatives to mental health appointments. Participants also highlighted the active role of family members in care, including attending appointments and reporting observed symptoms to healthcare professionals. Accordingly, they viewed family involvement in CBT sessions as both a source of motivation for individuals diagnosed with schizophrenia and a valuable source of information for therapists during assessment, particularly when individuals may not disclose or recognise certain difficulties.

*‘Usually, individuals diagnosed with schizophrenia or other mental illnesses don’t know what’s wrong with them, so when they visit an outpatient department, they are accompanied by a family member who informs the doctor of their symptoms and what they have observed about them. As a result, family members should be involved in the CBT intervention in order to assist the patient during the treatment stage’.*

(P8, Group 3)

Participants also reported that some family members held misconceptions about schizophrenia, including beliefs that the condition could not be managed effectively without medication. They observed that family members sometimes criticised individuals for non-adherence to prescribed medication without considering the underlying reasons and requested dose increases from psychiatrists to maintain clinical stability. Participants therefore recommended incorporating family education into the CBT intervention to improve understanding of schizophrenia, provide guidance on symptom management, and explain prescribed medications, potential side effects, and appropriate coping strategies.

*‘The majority of patient families are unaware of schizophrenia. They just see it as madness. They should receive information like diabetes education, which teaches them how to deal with the disease. The family plays an important role in the CBT intervention; they must participate as they will receive information on how to deal with the patient’.*

(P13, Group 3)

## 4. Discussion

The study found that mental health nurses in Saudi Arabia were generally supportive of delivering CBT interventions to individuals diagnosed with schizophrenia but identified barriers that could hinder implementation. These findings are consistent with qualitative studies examining healthcare practitioners’ perspectives on CBT for psychosis and other mental disorders in non-Western contexts [19,20,21,34]. Collectively, these studies highlight the contribution of family involvement to engagement with CBT and the importance of adequate training and supervision for healthcare practitioners. The present findings can also be considered alongside trial evidence for nurse-delivered CBT. In the randomised controlled trial by Turkington et al. [15], mental health nurses completed a 10-day intensive training programme delivered by CBT experts and, with weekly supervision, delivered six sessions over two to three months. The intervention was associated with significant improvements in symptoms and insight, high patient satisfaction, and, at one-year follow-up, better-maintained insight and fewer negative symptoms. These outcomes are encouraging for the Saudi context; however, our findings suggest that role clarity, organisational support, and culturally attuned training would be prerequisites for achieving similar implementation. Moreover, the present study highlighted the detrimental effects of overreliance on the medical model, characterised by a predominant focus on pharmacological treatment and limited availability of CBT for individuals diagnosed with schizophrenia [19,20,21,34].

Overall, the barriers and facilitators to nurse-delivered CBT can be considered at two levels: organisational and individual/family. At the organisational level, participants identified mental health nursing policy, dominance of the medical model, implementation within outpatient departments, supportive nursing management, and training. At the individual and family level, engagement of individuals diagnosed with schizophrenia and family involvement were considered important. Organisational barriers appeared interrelated, suggesting that addressing one may help mitigate others. For example, establishing a clearly defined scope of mental health nursing practice within policy, supported by nursing management, could enable mental health nurses to deliver psychosocial interventions such as CBT and reduce the dominance of a solely medical model. The absence of a well-defined scope of practice in Saudi mental health nursing has previously been identified as a barrier to nurses delivering such interventions [35]. Nursing management may therefore play a critical role in CBT implementation by advancing mental health nursing policy and facilitating effective interprofessional collaboration. Such efforts are important for enabling mental health nurses to provide high-quality, evidence-based care to individuals diagnosed with schizophrenia. A particularly notable finding was that some participants appeared to have internalised the medical model to the extent of endorsing coercive practices, including physical restraint, to enforce medication adherence. This finding is important because it illustrates how deeply a pharmacological, compliance-oriented paradigm may be embedded in everyday nursing practice and highlights a potential tension with the collaborative, recovery-oriented ethos of CBT. CBT for psychosis depends on a trusting therapeutic alliance, shared formulation, and respect for individual autonomy and personal goals; coercive approaches are inconsistent with these principles and may undermine engagement and trust. Preparing mental health nurses to deliver CBT in Saudi Arabia therefore cannot be a purely technical exercise. Training should also address attitudes and values and support a shift from a custodial, compliance-focused stance towards a recovery-oriented, person-centred approach. Reflective practice, clinical supervision, and values-based training are consequently likely to be important components of any CBT implementation programme in this context.

Viewed through an implementation-science lens, these barriers and facilitators extend beyond individual knowledge deficits. The apparent paradox, whereby nurses criticised the dominance of the medical model while simultaneously endorsing medication adherence and, in some accounts, coercive practices, may be understood as a product of professional socialisation within a strongly biomedical, psychiatrist-led hierarchy in which the mental health nursing role has historically been custodial and subordinate. Drawing on Normalisation Process Theory [36] and the Consolidated Framework for Implementation Research [37], implementation of nurse-delivered CBT would require attention to coherence (a shared understanding of the new therapeutic role), cognitive participation (commitment by nurses and the wider team), collective action (organisational sanction, workload redistribution, and training), and reflexive monitoring, alongside organisational readiness and redistribution of tasks across professional boundaries. From this perspective, the internalisation of compliance-oriented and coercive practices is not simply an individual attitudinal issue amenable to training, but a marker of the institutional culture and psychiatric hierarchy within which nurses practise. Addressing it is therefore likely to require concurrent policy, organisational, and cultural change alongside values-based education. The three themes also have broader interpretive significance. Nurses’ acceptance of CBT provides an encouraging basis for change and, in implementation terms, suggests emerging ‘coherence’ and ‘cognitive participation’: a shared sense that the intervention is meaningful and a willingness to participate. However, such willingness is unlikely to translate into practice without a clear and legitimate professional mandate. Similarly, role ambiguity and the dominance of the medical model reflect the wider organisational culture and hierarchy, described in these frameworks as the ‘inner setting’, rather than individual attitudes alone. Addressing these barriers therefore requires organisational-level changes that sanction and resource a new therapeutic role rather than education alone. Finally, the identified enablers, namely supportive nursing management, structured training, and family involvement, correspond to the organisational support and readiness that implementation frameworks regard as necessary for a new practice to become established and sustained.

Training emerged as a key facilitator of nurse-delivered CBT. However, mental health nurses in Saudi Arabia have limited opportunities for training in psychosocial interventions and mental healthcare [12,38,39], despite their willingness to provide such care. Ensuring access to structured training and ongoing supervision is therefore essential for the effective implementation of CBT for individuals diagnosed with schizophrenia.

Family involvement was also identified as an important facilitator of CBT delivery. Families play a substantial role in Saudi society, and caregiving is reinforced by religious obligations [40]. However, family involvement can also create challenges, particularly when relatives are overprotective or excessively involved [40]. For example, individuals diagnosed with schizophrenia in Saudi Arabia have reported that overprotective family members can invalidate their opinions and treat them ‘like three-year-olds’ [41]. Family involvement in CBT should therefore be aligned with the preferences of the individual.

These findings can be understood within the collectivist and family-centred context of Saudi society, in which the extended family, rather than the individual alone, is often a central unit of care and decision-making and caregiving is reinforced by religious and cultural obligations [40]. In this context, family members are often present at appointments, act as key informants, and mediate between individuals and services. This involvement creates opportunities to strengthen engagement, provide psychoeducation, and correct misconceptions about schizophrenia, but it may also marginalise the individual’s voice if autonomy is not actively protected. This configuration differs from the more individualistic assumptions that often underpin Western CBT, in which the therapeutic relationship is typically dyadic and centred on confidentiality. A culturally adapted CBT intervention for Saudi Arabia should therefore move beyond simply including family members towards a structured, negotiated model of involvement that harnesses the motivational and informational benefits of family participation while safeguarding confidentiality, preferences, and agency. This interpretation is consistent with the phase-one findings of this programme, in which individuals diagnosed with schizophrenia and their families identified family involvement as an important influence on engagement with CBT [22], and with wider evidence on culturally adapted psychosocial interventions in Arab and other collectivist contexts [19,20,21,28,34].

## 5. Strengths and Limitations of the Work

To the best of our knowledge, this is the first study to investigate mental health nurses’ perspectives on a proposed CBT intervention for individuals diagnosed with schizophrenia in Saudi Arabia. It is also the first study in this context to focus specifically on mental health nurses, whereas previous research has primarily explored the perspectives of psychologists and psychiatrists and has often excluded nursing professionals [19,20].

Despite these strengths, several limitations should be acknowledged. First, the study was conducted at a single urban tertiary mental health referral centre in Riyadh. The organisational context and availability of specialist services may differ from those in smaller, rural, regional, or private mental health services; consequently, the findings may not be transferable to all mental health settings across Saudi Arabia.

Second, although nursing managers did not select participants and interested nurses contacted the lead author directly, recruitment was facilitated through the head of nursing. This organisational recruitment process may have created perceived pressure to participate or concerns about expressing critical views.

Third, conducting focus groups among professional colleagues may have increased social desirability bias or inhibited dissenting perspectives, particularly during discussions of nursing management, psychiatrists, coercive practices, and organisational shortcomings.

Finally, because participation was voluntary, self-selection cannot be excluded; nurses with a greater interest in CBT may have been more likely to participate. However, the range of views expressed, including scepticism about the nursing role, criticism of existing practice, and divergent attitudes towards CBT, suggests that the sample was not limited to participants who were favourably predisposed to the intervention.

## 6. Conclusions

The findings indicate that mental health nurses expressed willingness to deliver CBT interventions to individuals diagnosed with schizophrenia. However, these findings reflect nurses’ perceptions and self-reported readiness; the study did not assess clinical competence, acceptability among individuals diagnosed with schizophrenia, or implementation and feasibility outcomes, and therefore does not establish that nurse-delivered CBT is ready for routine implementation. Rather, the study identifies perceived barriers and facilitators that subsequent phases of the programme will need to address. Many participants lacked clarity about the mental health nursing role and reported insufficient knowledge and skills to deliver CBT. Effective implementation will therefore require collaboration among mental health nurses, policymakers, families, individuals diagnosed with schizophrenia, psychiatrists, and other relevant stakeholders to clarify mental health nursing policy and ensure adequate training opportunities. The next stage of the programme will involve seeking funding and continuing to apply the Medical Research Council framework for complex interventions, with a focus on intervention modelling [24]. This phase will include development of a training manual for mental health nurses, followed by a feasibility evaluation of the acceptability, deliverability, and implementation of the proposed CBT intervention.

## Figures and Tables

**Table 1:** Demographic and professional characteristics of participating mental health nurses.

Participant No.	Years Since Qualification	Professional Grade	Qualification	Gender
P1	2	Staff mental health nurse	Bachelor’s degree	Male
P2	7	Staff mental health nurse	Diploma degree	Male
P3	5	Staff mental health nurse	Bachelor’s degree	Male
P4	15	Staff mental health nurse	Bachelor’s degree	Male
P5	7	Staff mental health nurse	Bachelor’s degree	Female
P6	13	Charge mental health nurse	Bachelor’s degree	Female
P7	8	Staff mental health nurse	Diploma degree	Male
P8	12	Charge mental health nurse	Bachelor’s degree	Female
P9	11	Staff mental health nurse	Diploma degree	Male
P10	7	Staff mental health nurse	Diploma degree	Female
P11	10	Charge mental health nurse	Master’s degree	Female
P12	10	Staff mental health nurse	Bachelor’s degree	Female
P13	5	Charge mental health nurse	Bachelor’s degree	Male
P14	10	Staff mental health nurse	Bachelor’s degree	Female
P15	7	Charge mental health nurse	Diploma degree	Male
P16	18	Staff mental health nurse	Bachelor’s degree	Male
P17	11	Staff mental health nurse	Bachelor’s degree	Female
P18	11	Staff mental health nurse	Bachelor’s degree	Female
P19	11	Staff mental health nurse	Diploma degree	Female
**Summary (*N* = 19)**	**Range 2–18; mean 9.5; median 10**	**Staff mental health nurse, 14 (74%); charge mental health nurse, 5 (26%)**	**Bachelor’s degree, 12 (63%); diploma, 6 (32%); master’s degree, 1 (5%)**	**Female, 10 (53%); male, 9 (47%)**

Note: Bolds—Summary.

**Table 2:** Thematic framework derived from mental health nurses’ focus groups.

Theme	Sub-Theme	Representative Quotation	Interpretation
**Acceptability of the CBT intervention**	Key elements of the CBT approach	*“These components, in my opinion, will help people who have schizophrenia deal with their symptoms.”* (P6, Group 2)	Nurses viewed the proposed CBT components as relevant and beneficial, indicating genuine openness to the intervention and its structured, relational approach.
**Challenges to CBT implementation**	Constraints within mental health nursing policies	*“As a mental health nurse in our outpatient department, I do not have any role to play as a therapist. My role is to just take vital signs… If CBT isn’t an essential part of the job description, I’m not obliged to do it…”* (P5, Group 1)	The absence of a clear nursing policy confines nurses to basic tasks and leaves no defined therapeutic role, representing a structural rather than an individual barrier.
	Predominance of the medical model	*“…the doctors play the most important role… they do not refer them to a psychologist but instead prescribe medications… This is an incorrect approach that has been practised for years.”* (P7, Group 3)	The dominance of a medication-focused, psychiatrist-led model marginalises psychological interventions and constrains the space for nurse-delivered CBT.
**Enablers of CBT implementation**	Mental health nursing management	*“…the nursing administration… have the authority to add the CBT intervention to the nursing policy and nominate the person who will deliver the intervention…”* (P5, Group 1)	Nurses identified nursing management’s authority to formalise CBT within policy as a key organisational enabler.
	Availability of training opportunities	*“As a mental health nurse, I should have the required training to offer the CBT intervention. We have many departments; we are ready, but we lack the tools…”* (P15, Group 3)	Structured training and supervision were viewed as prerequisites; willingness needed to be matched by capacity building.
	Active involvement of family members	*“The majority of patient families are unaware of schizophrenia. They just see it as madness…”* (P13, Group 3)	Family engagement and education were viewed as central to CBT’s success, reflecting the relational and culturally embedded nature of care.

Note: Italics—Participant quotations.

## Data Availability

The data presented in this study are available on request from the corresponding author. The data are not publicly available due to privacy and ethical considerations involving human participants.

## Supplementary Materials

The following supporting information can be downloaded at: https://www.mdpi.com/article/10.3390/healthcare14172852/s1, File S1. Standards for Reporting Qualitative Research (SRQR) Checklist [26]. File S2. Focus Group Topic Guide for Mental Health Nurses.

## Abbreviations

The following abbreviation is used in this manuscript:

CBT	cognitive behavioural therapy
