# Supplementary material for: Mental Health Nurses’ Perspectives on Cognitive Behavioural Therapy for Individuals Diagnosed with Schizophrenia in Saudi Arabia: A Qualitative Study
Source: Healthcare (Basel). 2026 Sep 4;14(17):2852. doi: 10.3390/healthcare14172852 (PMC13566609; doi:10.3390/healthcare14172852)
Supplement: Supplementary file 1 [file healthcare-14-02852-s001.zip › Supplementary File S2-Focus Group Topic Guide for Mental Health Nurses.pdf]

Supplementary File S2: Focus Group Topic Guide for Mental Health Nurses

Warm up

Can you tell us your name and position, please?

Introduction

Before participants were asked about the CBT program, a brief explanation of each component was introduced as follows:

Engagement and assessment

The aim of this stage is to build a rapport, an important therapeutic relationship between the therapist and patient, and to collect information by using assessment tools to understand the problems and experiences that cause distress to the patient. During this phase, the therapist should be listening carefully to the patient's concerns and empathise with them. In addition, the therapist should be describing the process of the intervention to the patient and answering their questions.

Psychoeducation:

This phase usually follows the engagement phase. The therapist, in this phase, is providing information about schizophrenia, including its causes and descriptions of various symptoms. The aim of the sessions is not just to teach the participant facts about schizophrenia, but also to help the participant understand the nature of schizophrenia and how different factors can increase susceptibility to schizophrenia.

Working with psychotic symptoms

This phase includes working with delusions, hallucinations and negative symptoms, and coping strategies. The therapist helps patients to understand the link between their beliefs and thoughts, and how these can impact their feelings and behaviour. Following this, the therapist can help the patient to change disruptive delusions and beliefs by developing alternative explanations in order to reduce their distress. In addition, the therapist will educate the patient about coping strategies in order to help them better manage their symptoms and enhance personal recovery. For example, teaching a patient who has hallucinations to use ear plugs and headphones to distract from or stop the voice.

Medication management

In this phase the therapist will discusses the advantages and disadvantages of medication. If the patient is taking medication, the therapist will discuss the side effects of the medication and how they can help the patient to manage their side effects. The therapist will explain how to take medication effectively.

Relapse prevention

In this phase the therapist will help patients understand the early signs of relapse and how they can reduce the chances of relapse. In addition, the therapist will help the patient to identify specific triggers of relapses and enable them to develop a relapse-prevention plan.

Main questions

1- What are your first impressions of the programme?
2- What could be the barriers to delivering this programme?
3- How would you make this programme a success?
4- What training do you need to deliver this programme?
